# Circadian Rhythms in Socializing Propensity

**DOI:** 10.1371/journal.pone.0136325

**Published:** 2015-09-09

**Authors:** Cheng Zhang, Chee Wei Phang, Xiaohua Zeng, Ximeng Wang, Yunjie Xu, Yun Huang, Noshir Contractor

**Affiliations:** 1 Department of Information Management and Information Systems, Fudan University, Shanghai, China; 2 College of Business, City University of Hong Kong, Hong Kong SAR, China; 3 School of Communication, Northwestern University, Evanston, Illinois, United States of America; East China University of Science and Technology, CHINA

## Abstract

Using large-scale interaction data from a virtual world, we show that people’s propensity to socialize (forming new social connections) varies by hour of the day. We arrive at our results by longitudinally tracking people’s friend-adding activities in a virtual world. Specifically, we find that people are most likely to socialize during the evening, at approximately 8 p.m. and 12 a.m., and are least likely to do so in the morning, at approximately 8 a.m. Such patterns prevail on weekdays and weekends and are robust to variations in individual characteristics and geographical conditions.

## Introduction

“Human existence is social through and through.” ([1], p.3)

“In the form of time is to be found the form of living.” ([2], p. 129)

Socializing is a basic human need, along with movement, love, and dignity [1, 3]. A foundation of socializing is mingling with people and establishing connections to build social networks. Through social connections, people gain access to important resources, including job opportunities, knowledge and advice, and financial and emotional support [4].

Previous research has attempted to uncover the reasons for and the conditions in which people socialize by forming new connections. A typical focus has been the principle of homophily, or the idea that “similarity breeds connection” [5]. The tenet is that people tend to positively evaluate those who share similar socio-economic conditions, backgrounds, interests, and behaviors with them, which leads to a psychological predisposition to form ties with similar others [5]. Individual characteristics aside, research has also examined contextual conditions such as geography and has found that people who are proximate to one another are more likely to form social connections than those who are distant apart [6]. Time [7], another fundamental contextual condition of human activity, has been neglected in the literature, although what we do in everyday life is deeply interwoven with time [2].

Recent literature has shown that there are strong temporal regularities in human behavior. For instance, [8] observes that mobile phone users’ mobility patterns have a high degree of temporal regularity in addition to spatial regularity. Likewise, [9] indicates that people typically wake up in a good mood that deteriorates as the day progresses, with negative moods reaching peaks at night and in the early morning. Furthermore, [10] shows that Twitter users are most likely to tweet about problem drinking on Friday, Saturday, and Sunday from 10 p.m. to 2 a.m. Although these studies underscore time as a relevant dimension that conditions human behavior, it remains unclear whether and how human socializing varies temporally.

A possible reason for the lack of attention to the temporal dimension of socializing is the challenge of recording precisely *when* people establish social connections and computing the *probability* of them doing so in all of their social activities during a certain period. The emergence of social media may help alleviate this challenge. As noted by [11], social media offers the opportunity, for the first time, to observe human behavior and interactions in real time for two reasons. First, human interactions leave microscopic traces on social media platforms that are comprehensively recorded and time-stamped [12]. Second, many activities of everyday life are now occurring online. Despite certain differences between the online and offline environments (e.g., the lifting of geographical constraints), we remain the same person online or offline, with similar needs to fulfill. For instance, we exchange information with friends on Twitter to obtain news updates, we engage in expertise sharing with fellow professionals to gain recognition within the community, and we meet and connect with new people in virtual worlds to obtain instrumental and emotional support [11]. By contrast to the self-reporting methodology applied in offline studies, the objective measures afforded by social media are neither prompted by an experimenter nor recollected after the event. Instead, they are directly recorded by a server in real time from people who are not interrupted in their usual course of behavior. Therefore, the data collected from social media are less vulnerable to memory bias and experimenter demand effects, and have increasingly been employed in recent years to study human behavior (e.g., [9, 10, 13, 14, 15]).

This study employs data from the virtual world of a popular massive multiplayer online role-playing game (MMORPG) called Dragon Nest. The online gaming environment is a three-dimensional space that simulates a real-world social environment in which people perform various tasks while meeting and interacting with other participants via their avatars (virtual representations of themselves), making it suited for studying human behavior (e.g., [16, 17, 18]). As with online social networking websites, the platform provides broad functionalities to enable participants to build their social networks. Our data include 1-million active game players from diverse demographic backgrounds and their friend-adding behavior during a three-month period from January 1 to March 31, 2011. More information details about the game and the dataset is provided in the S1 File.

Compared to data from other social media platforms, such as online communities, social networking websites, and Twitter, which have been widely employed for human behavioral research (e.g., [9, 10, 14]), our data afford two advantages for studying socializing behaviors. First, information about a person’s latent connections is typically absent in these other platforms. Researchers do not know what users actually see at any particular moment (how many others are around to possibly establish social connections) when they decide to make new connections. This information is crucial to measure a person’s propensity to make social connections; without this information, it is impossible to determine whether a user has made more social connections simply because he has met more people than others or because he is more inclined to do so at that particular moment. This difficulty is alleviated with our data from the online environment that allows latent connections to be observed. Second, compared to primarily textual-based platforms such as Twitter, the three-dimensional virtual environment of the online game constitutes a more realistic environment for socializing that closely parallels the real world, in which people are able to move around, see one another, engage in activities together, and make decisions to establish social connections with others (or not).

## Method and Results

**Fig 1:**
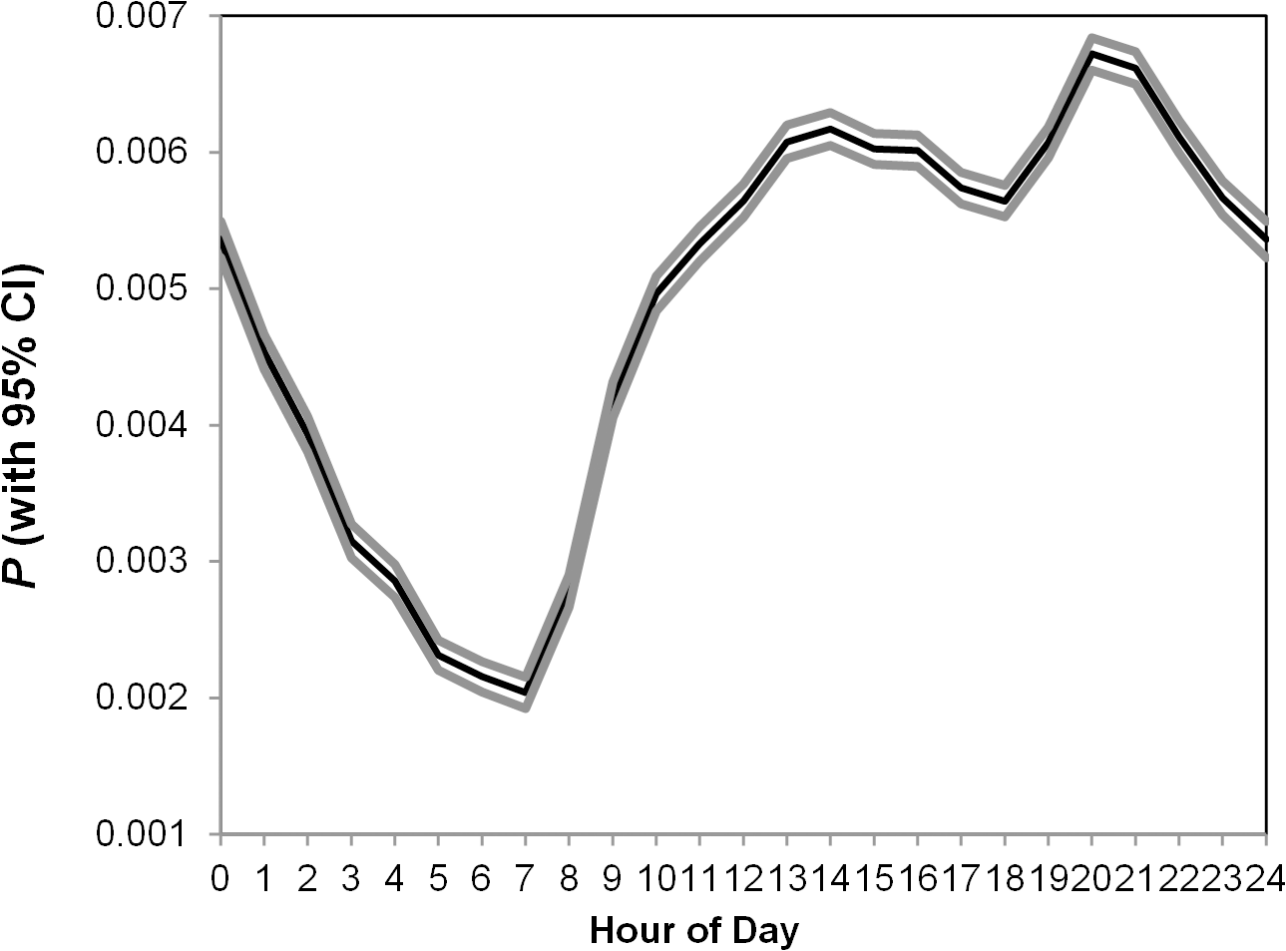
Probability of Making Social Connections by Hours of the Day.

Utilizing the online game data, we observed 120,142,105 pairs of social connections initiated during the data period. In accordance with the company’s agreement, all data was provided and analyzed anonymously, and do not contain any identifiable personal information (including the IP addresses employed which are dynamic and only fixed to a specific area). Users’ behavioral information was also analyzed at an aggregated level. Fig 1 below depicts the general probability of making social connections by hour of the day. The probability was lowest in the morning at approximately 7 am (*Mean* = 0.0020, *SD* = 0.0001); after that, it increased sharply and reached its peak during the night, at approximately 8 p.m. (*Mean* = 0.0067, *SD* = 0.0001; *t* (460,015) = 55.61, *P* < 0.001).

Conceptually, when the general probability in two hours are different, there can be two possibilities: individual players had different probabilities of making social connections in these two hours (i.e., within-individual variations; e.g., an individual player tended more to make friends at time t compared to time t+1), or different composition of players were present in the two hours, such as the players present at one hour are more active than those present at another hour (i.e., between-individual variations; e.g., socially active players are more likely to be present in the game environment at time t compared to time t+1). For each individual player, we first calculated his or her baseline probability (*BSP*), i.e., the average probability of making social connections across all hours (see Fig A in S1 File for the distribution of the baseline probabilities of the players). Then, for a given hour, we decomposed the pattern into within-individual probability (*WP*), which indicates the individual players’ deviation from their own baseline probability, and between-individual probability (*BP*), which averages the baseline probabilities of the players present in that hour (the details are in the S1 File). Because our purpose is to uncover the temporal patterns or circadian rhythms in individuals’ propensity to make social connections, we based most of our analyses on *WP*, which is consistent with [9]. However, we also checked *BP* because it might contribute to differences in the general probabilities of making social connections.

**Fig 2:**
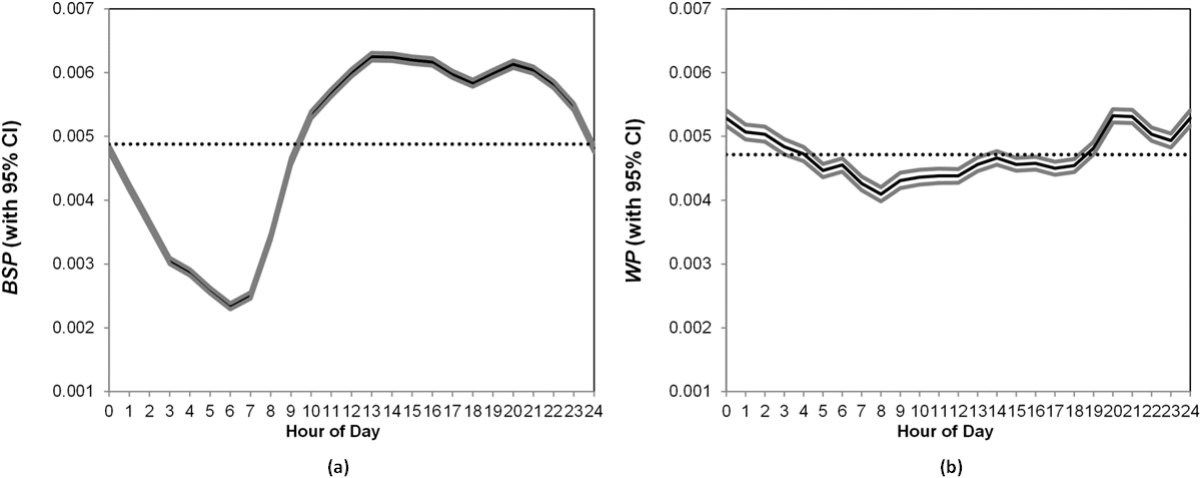
(a). Between-individual Probability (BP), (b). Within-individual Probability (WP). * Dotted lines indicate the mean level.

The between-individual pattern (Fig 2A) indicates that players at different times varied in their inclination to make social connections. For example, people who played the game at 6 a.m. (*Mean* = 0.0023, *SD* = 0.0099) were least likely to add friends, whereas people who played the game at 1 p.m. were most active in adding friends (*Mean* = 0.0063, *SD* = 0.0170; *t* (489,344) = 101.93, *P* < 0.001).

With respect to within-individual variations (Fig 2B), although the pattern is flatter, it continues to exhibit a statistically significant variation across the hours of a day (*F* (23, 6,432,744) = 38.43, *P* < 0.001). The probability of making social connections has a trough at 8 a.m. (*Mean* = 0.0041) and peaks at 8 p.m. and 12 a.m. (both *Means* = 0.0053; *P* < 0.001), which indicates that individual players had a higher inclination to make new social connections at night than in the morning.

To rule out alternative explanations, we examined factors that could potentially contribute to the observed pattern such as number of players in the game. Results indicate that, regardless of how many other people are present in the surrounding environment, the circadian rhythm of people’s propensity to form social connections persists (see Fig B in S1 File). More details are provided in the S1 File.

**Fig 3:**
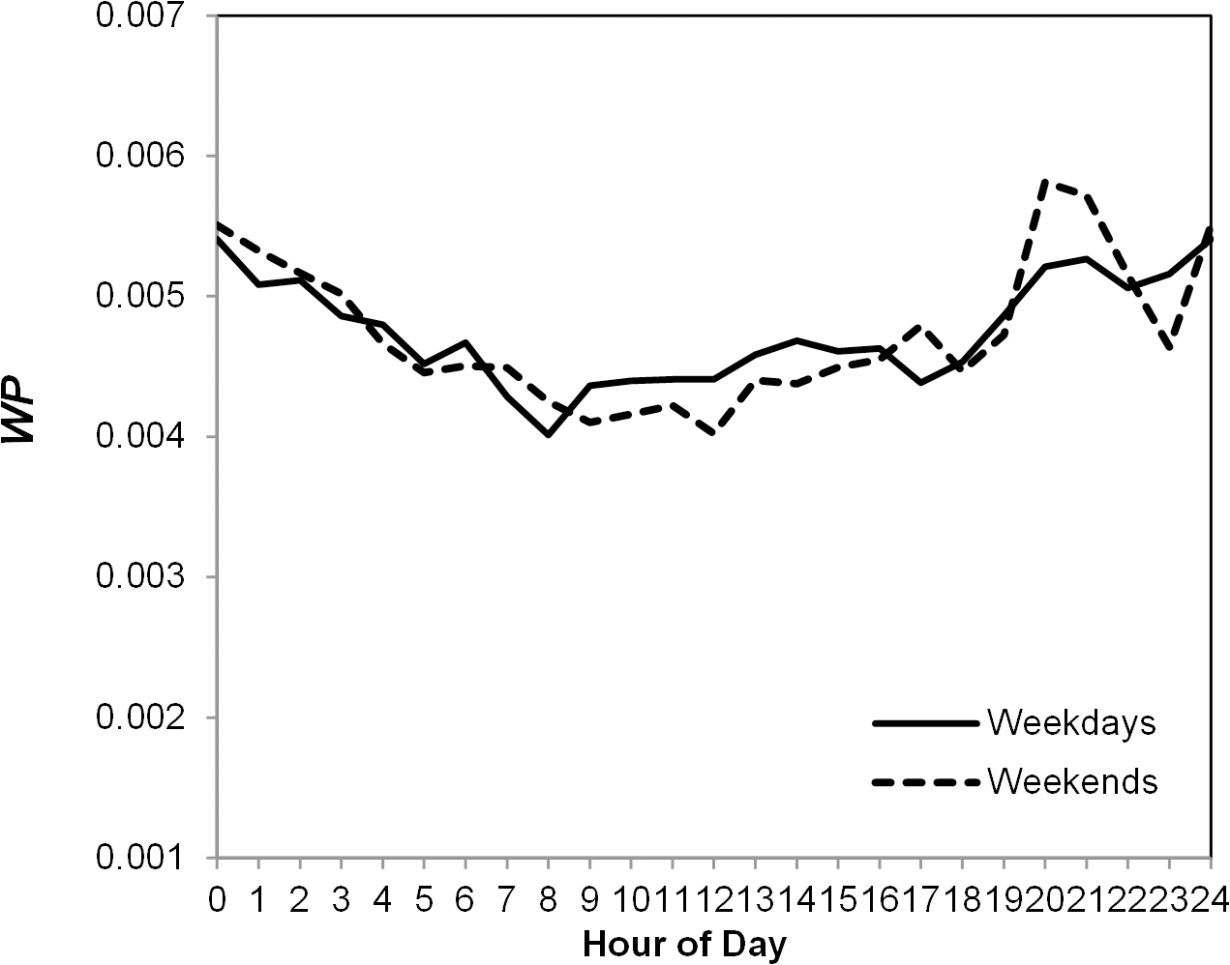
Probability of Making Social Connections by Weekends/weekdays.

We also plot the patterns for weekdays and weekends in Fig 3. Both curves exhibit a similar cycle and have significant variations by the hour of the day (*F* (23, 5,407,174) = 30.16, *F* (23, 3,070,762) = 31.71, both *Ps* < 0.001) despite slight differences (for instance, an additional peak can be observed at 5pm during the weekends, which may be due to the fact during the weekends, people do not need to spend their time in public transportations to go back home). This finding seems to suggest that the propensity to socialize could be affected by biological clock and sleep—because people usually wake up later on the weekends, their inclination to make social connections also begins later, but sleeping for a longer period makes them even more active during the peak hours especially at night.

Because the temporal patterns in the propensity to make social connection of individuals can be very different, we classified the players into groups based on several characteristics including their level of possession (wealth) and gaming experience. For example, we classified the players based on their level of possession in real life (at a group level, as confidentiality issue forbids us from obtaining information about individual income), as proxied by the GDP level of their location, and their level of possession in virtual-world, as indicated by whether they made purchase in the game. Similar circadian rhythm in the probability of making social connections was observed (see Fig C and Fig D in S1 File). More details are discussed in the S1 File.

Other robustness checks conducted including whether the circadian rhythm observed is tied to players’ game achievement level attained (see Fig E in S1 File), number of days being in the game after registration (see Fig F in S1 File), and number of friends they have (see Fig G in S1 File). Similar patterns are observed; refer to S1 File for more details of these robustness checks.

As a further assessment of the observed circadian rhythm in people’s formation of social connections, we applied the Social Rhythm Metric (SRM) analysis which is intended to capture the extent of regularity or rhythm in people’s daily activities [19]. In the original SRM study, subjects completed the measure in the evening before going to bed to track the timing of 15 specific activities (e.g., waking up, taking meals, etc.) performed over the day. Consistencies in the timing of these activities indicate the stability of an individual's daily routine. In our study, if a peak or trough matches the general pattern (within an interval of ± 1 hour), then it is considered a “hit”. Table 1 presents a summary of the SRM analysis results. The results show that the majority of the peaks (54%) hit within the range of one hour, though the hit rate for troughs is lower than 50%. The results are generally consistent with those reported in the literature that indicates stability in human behavior, with hit rates ranging from 0.43 to 0.62 [20, 21]. Thus, the results generally support a high stability of the circadian rhythm of people’s formation of social connections over the 3-month period.

**Table 1:** Summary of the SRM Results.

	± 1 hour	
	Hit/Miss	Hit Percentage
Peaks (9 p.m. &12 a.m. on weekdays; 8 p.m. & 12 a.m. on weekends)	42/36	54%
Trough (8 a.m. on weekdays; 12 p.m. on weekends)	36/42	46%

Logically, the circadian rhythm of people’s socializing propensity should demonstrate geographical deviations corresponding to time zone changes. That is, people in the Western districts may have peaks and troughs that appear later than people in the Eastern districts. Unlike many countries, China enforces an official unified time of Beijing (GMT+08:00) across the country, although its geographic homeland covers between GMT +09 and GMT+05. It may be that this official time reconciles with the natural local time and exhibits some notably compromised behavioral results. Balancing population distribution, we longitudinally divided all players into two regions: the East region, which covers the GMT+09 and GMT+08 time zones, and the West region, which covers the GMT+07 to GMT+05 time zones. Each region consists of several provinces (refer to Table A in S1 File). Fig H in S1 File depicts the patterns for two different time zones. The two curves exhibit similar rhythmic patterns and, notably, have the same peak (12 a.m.) and trough (7 a.m.). This result seems to suggest that the effect of political, official time has dominated the effect of local, natural time on the rhythmic cycle of people’s propensity to make social connections.

## Discussion

The findings from this study must be interpreted in light of its limitations. Unlike survey-, interview-, or laboratory-based studies, we have the advantage of detailed, large-scale data to conduct group-level analyses, but we do not have knowledge about the underlying psychological mechanisms that explain people’s choices in making social connections. Nevertheless, the clear circadian rhythm revealed in the formation of social connections after controlling for potential influences has several important implications. To social scientists, the temporal dynamics in people’s socializing propensity calls for additional care in conducting social science research. Specifically, social scientists must be sensitive to the timing of administering surveys or experiments that study people’s social perceptions and associated behavioral consequences. If a study is administered entirely within a certain timeframe during which a peak or trough of socializing propensity occurs, socially biased results could ensue. Ideally, data collection should be spread across different times of a day to obtain a more comprehensive coverage of possible social states and to rule out potential temporal influences. For policy makers, the circadian rhythm may help determine the best timing to employ (or avoid) for socializing new members into a community. In the organizational context, socializing new employees into the existing employee community may foster team building and the transfer of tacit knowledge [22]. In the societal context, socializing new residents with an existing resident community may help to cultivate a more harmonious and vibrant community [23]. Doing so at the “right” times may lead to better outcomes. The same principle may be applied to other scenarios, such as charity and political mobilizations, in which promoting socialization at the right time is important for obtaining support.

The clear circadian rhythm of people’s socializing propensity may be linked to a number of possible factors that call for future research. First, research has shown that the release of hormones such as oxytocin may promote a higher trust in others that may in turn improve socialization [24], and that their release demonstrates circadian rhythms [25]. Second, positive moods have been found to incline people to feel more social [26], and that people’s moods vary by hours of a day [9]. Still possibly, it could be the release of certain other hormones that regulate moods or affective states [27], which in turn influence human socialization. Future research may investigate if one of these factors, or a combination of them working together or in a chain, that contribute to the circadian rhythm of human socializing behavior.

Furthermore, while our robustness check shows that the probability of making social connections is relatively independent from the number of people who were in the virtual game environment, the comparison offers an additional interesting insight that deserves further investigation. Specifically, during the night and wee hours when the number of players were decreasing in number (in particular, from about 9 p.m. to 8 a.m.), we could observe a peak of people’s socializing tendency that occurred at 12 a.m., which gradually decreased over the night times but still higher than that during the bright day times on average (e.g., from 8 a.m. to 6 p.m., compared to 12 a.m. to 3 a.m.). This may hint that during the late night and wee hours when there are not many people around, it may be cozy for people to engage in social activities akin to people socializing in a small pub during those hours. It will be interesting for future research to investigate the multitude of factors, both physiologically and psychologically, that contribute to the clear circadian rhythms in people’s socializing tendency. We hope this work can serve as a foundation that stimulates future research in this direction to enrich our understanding about the temporal aspect of this fundamental human behavior.

## Supporting Information

S1 File Fig A. Distribution of the Probability of Making Social Connections. Fig B. Comparison between Within-individual Probability (WP), Number of Players Online, Number of Players Engaging in Game Activity (PVE), and the Residual of WP on PVE. Fig C. Probability of making social connections by GDP level. Fig D. Probability of making social connections by online purchase. Fig E. Probability of making social connections by game achievement levels. Fig F. Probability of making social connections by number of days being in the game after registration. Fig G. Probability of making social connections by number of friends. Fig H. Probability of making social connections by time zones. Table A. List of time zone divisions. (ZIP)
