# Supplementary material for: Circadian Rhythms in Socializing Propensity
Source: PLoS One. 2015 Sep 9;10(9):e0136325. doi: 10.1371/journal.pone.0136325 (PMC4564240; doi:10.1371/journal.pone.0136325)
Supplement: S1 File — (ZIP) [file pone.0136325.s001.zip › S1 File/S1 File.docx]

S1 File - Supporting Information

**Materials and Methods**

1. **Game Data Background**

This study employs data from the virtual world of a popular massive multiplayer online role-playing game (MMORPG) called Dragon Nest. The data contains game players’ behavioral information that was recorded objectively in real time by the game system. Following the term-of-use agreement signed by the company and the authors before this project, the company provided anonymous records of a random set of game players in data table format, masking any identifiable personal information with random generated id numbers. Although various types of game players’ behavioral information are recorded across several data tables in the game system, unique random generated user ids can be used to track and aggregate game players’ behaviors. Game players agreed the company to collect and analyze their behavior data in a way that does not disclose their personal information without any further noticing or written consent before playing the game.

The online gaming environment is a three-dimensional virtual space that simulates a real-world social environment in which people perform various tasks while meeting and interacting with other participants via their avatars (virtual representations of themselves). To add another player as a friend, an individual can click on that player’s profile picture or use the “add friend” feature in the game menu. A player can unilaterally add another player to his or her friend list, and the latter is notified immediately. Being friends allows the players to communicate easily, to share news and information (such as tips about playing the game), and to complete tasks together. Our data include 1-million active game players from diverse demographic backgrounds and their friend-adding behavior during a three-month period from January 1 to March 31, 2011 (eighty days in total; ten days are missing as a result of server maintenance). We have the following three sets of information: (a) every instance of friend adding between two players during the period and the time-stamp of when it occurred (date and time); (b) each player’s latent connections (potential friends), i.e., the other players whom each player has met in the virtual environment when accomplishing game mission together (denoted by PVE, or player-versus-environment), and with whom he or she has had a chance to establish a connection during the period; and (c) each player’s characteristics, such as game achievement level and IP address[[1]](#footnote-1). Although we do not have demographic data on the players in the sample for this study, a survey of 19,013 sampled players conducted by the firm in early 2013 shows that 30.2% of the players were between the ages of 18 and 25, 47.6% of the players were between 26 and 33 years of age, 20.6% were older than 33, and the remainder (1.6%) were younger than 18. In terms of occupation, 11.0% were students or unemployed, 24.8% were self-employed, and the rest (64.2%) were employed full time. These statistics indicate that the community that plays the game represents a relatively diverse population.

1. **Measurement of the Probability of Forming Social Connections**

We computed the probability of forming social connections by individual players in the following manner: for each player in a given hour, we counted the number of his or her latent social connections (*PFnd*) and the number of social connections he or she made (*Fnd*) during this hour and then calculated the following probability:

,

where *U* and *U* are all the players in our sample, *h**H* and *H* = {0…1919} (assuming 0-23 for day 1, 24-46 for day 2, etc.). Latent social connections refer to the people a player encountered when accomplishing a game mission together. For example, in a given hour, if a player played a game mission together with three strangers, then the player has three latent social connections. If he or she adds one of them as a friend, then *P(u,h)* is 0.33 for this given player-hour. Note that if a player has not logged into the game in an hour or has logged in but encountered no potential/latent social connections, we coded the probability for that player-hour as missing. We examined how the probability varies as a function of the hour of the day and the average *P(u,h)* for each given hour of a day (0-23 hour) over the three-month period.

To measure within-individual variations, for each user, we first calculated his or her baseline probability (*BSP*) (averaging *P(u,h)* across all hours for this individual):

.

Next, the within-individual measure (*WP*) was computed as follows:

.

The within-individual measure represents the individual player’s deviation from his or her own baseline probability, which allows us to focus on the player’s rising and falling socializing behavior by the hour of the day. The last term is the grand mean across all players over all hours (in our case, 0.004733; all player-hours are equally weighted). Adding this term does not change the shape of the pattern; it only shifts up on the y-axis, which provides easier interpretations.

The between-individual variation (*BP*) was computed as follows:

,

where *U(h)* refers to all players who were playing the game during an hour *h*. A higher *BP(h)* indicates that the players in that hour were more active players in terms of their general probabilities of making social connections.

To examine within-individual variations, we must observe that a player made social connections more than once. This filtering condition led to a selection of 732,426 players who had logged into the game on at least three days during the three-month period. Of these players, 7% logged into the game but did not participate in any game activity with others (i.e., zero latent social connection); thus, they were excluded from our analyses. This process led to a sub-sample of 679,213 players.

Within this sample, 19% of the players made at least one social connection during the data period (i.e., *BSP(u)* is non-zero). Fig. A below depicts the distribution of the baseline probabilities of these players; 99% of the players had a baseline probability of making social connections lower than 0.20.

**Fig. A. Distribution of the Probability of Making Social Connections.**

1. **Robustness Check**

To rule out alternative explanations, we examined factors that could potentially contribute to the observed pattern. One potential factor is the number of players in the game, which varies by the hour of the day and represents external opportunities in the environment for people to form social connections. When there are more players in the game, people can engage in game activities with more players, which may increase their tendency to form social connections. To examine this possibility, we plotted the graph of the within-individual probability against the number of players online and players engaging in game activities (team play for mission completion, denoted by “player-versus-environment” (PVE)) (see Fig. B). For ease of comparison, we rescaled the two numbers such that they fit into the same graph; then, we examined the correlations among the measures. The within-individual probability was only weakly correlated with both the number of players online (*r* = 0.0060) and the number of players engaging in game activities (*r* = 0.0047), which indicates the relative independence of the pattern from the potential influence of the number of players online. In other words, regardless of how many other people are present in the surrounding environment, the circadian rhythm of people’s propensity to form social connections persists.

Furthermore, we regressed within-individual probability on number of players engaging in game activity (because it is highly correlated with the number of players online, we skipped the latter). Fig. B shows that the residual continues to exhibit the rhythmic changes over time in a day (*F* (23, 6,432,720) = 32.29, *P* < 0.001). For example, the peak (12 am) (*Mean* = 0.0006, *SD* = 0.0311) remains significantly higher than the trough (8 am) (*Mean* = −0.0004, *SD* = 0.0248; *t* (448,067) = 12.44, p < 0.001).

**Fig. B. Comparison between Within-individual Probability (WP), Number of Players Online, Number of Players Engaging in Game Activity (PVE), and the Residual of WP on PVE.**

Below, we examined whether the temporal patterns of socializing propensity vary across individuals. We first examined whether or not socializing propensity is associated with a player’s level of possession (wealth), both in real-world (at a group level proxied by the GDP of the province where they are located) and in virtual-world (indicated by whether they made purchase in the game). Because we did not observe individual player’s actual income level in real-life, we approximated this at a group level based on the GDP level of their location. Based on the GDP level of each player’s location, we classified the players into three groups: high-, middle-, and low-GDP. The within-individual pattern (Fig. C) indicates that socializing propensity varied by hour of the day for all the three GDP groups (all Fs > 5.24, all Ps < 0.001). For example, in the high GDP areas, the probability of making social connections has a trough at 8 a.m. (Mean = 0.0041) and peak at 12 a.m. (Mean = 0.0078; t = 19.35, P < 0.001). The other two GDP areas exhibited similar patterns.

**Fig. C. Probability of making social connections by GDP level**

For level of possession in the game, we used players’ purchase made in the game to represent his or her level of possession in virtual-world. In our data period only 6% of the players made a purchase and the rest did not. We plot the temporal patterns of them in Fig. D. Again, the within-individual pattern indicates that socializing propensity varied by hour of the day regardless whether or not the player made a purchase (both Fs > 21.61, Ps < 0.001). For example, for the players who made a purchase, the probability of making social connections has a trough at 8 a.m. (*Mean* = 0.0023) and peaks at 9p.m. (*Mean* =0.0063; t = 9.58, P < 0.001) and 2a.m. (*Mean* = 0.0069; t = 8.47, P < 0.001).

**Fig. D. Probability of making social connections by online purchase.**

In addition, we examined whether or not socializing propensity is associated with a player’s gaming experience. Players with more gaming experience may be more confident in establishing connections with others. We focused on two indicators of gaming experience, game i.e., achievement level and number of days staying in the game after registration. First, in the game, as a player completes more pre-determined goals, he or she progresses through levels that are numbered from 1 to 40. We classified the players into a low-level group (levels 1-12) and a high-level group (levels 13-40), and each group accounted for approximately 50% of the players. In terms of the baseline probability, the high-level group had a significantly higher probability of making connections than the low-level group (*Mean* = 0.0055 vs. *Mean* = 0.0041; *t* (679,190) = 29.63, *P* < 0.001), which indicates that players with higher achievement levels had a higher inclination to make social connections. We further examined whether the two groups differed in their temporal pattern, or circadian rhythm, of making social connections. Fig. E shows that the high-level group demonstrated a greater variation in probability: it was significantly lower than that of the low-level group during the trough period (from 2 am to 8 am; all *Ps* < 0.05) but significantly higher during peak hours (8 pm to 10 pm; all *Ps* < 0.05). The results indicate that players with higher achievement levels were more selective in the timing of establishing social connections with others, whereas junior players were making friends at a more even rate during the day. Nonetheless, a clear circadian rhythm in the probability of making social connections was observed in both groups (e.g., the probability of the low-level group varied significantly by the hour of the day, *F* (23, 2,804,638) = 20.04, *P* < 0.001), which is consistent with the general pattern we observed previously. Similarly, based on the number of days players have been staying in the game after registration, we used the median (i.e., 50 days) to split the players into two groups. The temporal patterns (Fig. F) coincide with Fig. E. The players who registered for longer time period exhibited a greater variation in socializing probability than those who registered for a short time period. Nevertheless, the probability of both groups varied significantly by hour of the day (both *F*s > 4.55, Ps < 0.001).

**Fig. E. Probability of making social connections by game achievement levels.**

**Fig. F. Probability of making social connections by number of days being in the game after registration.**

Another interesting question is whether or not the temporal pattern of socializing propensity is associated with the number of friends that individuals have. We focused on individuals who have added at least one friend by the end of our data period, which accounts for 29% of the players. The median number of friends for these players is 10. Fig. G shows the temporal patterns after the median-split. Regardless of the number of friends they have, similar circadian rhythm of making social connections were observed (both *F*s > 11.30, Ps < 0.001). For example, for the players who have fewer than 10 friends, the probability of making social connections has a trough at 7 a.m. (*Mean* = 0.0017) and peak at 8p.m. (*Mean* =0.0063; t = 10.49, P < 0.001).

**Fig. G. Probability of making social connections by number of friends.**

Since our data contain the IP address of each player, this allows us to examine the potential effect of time zone changes. Balancing population distribution (because most Chinese people live along the east coast), we longitudinally divided all players into two regions: the east region, which covers the GMT+09 and GMT+08 time zones, and the west region, which covers the GMT+07 to GMT+05 time zones (see Table A that shows the different time zones and the corresponding provinces; note that the percentages of game population in the different time zones closely resemble those of the national population). Fig. H depicts the patterns for the two different time zones.

**Fig. H. Probability of making social connections by time zones.**

1. **Social Rhythm Metric (SRM) Analysis**

The SRM is intended to capture the extent of regularity or rhythm in people’s daily activities. Consistencies in the timing of activities indicate the stability of an individual's daily routine. If the timing of an activity on a given day occurs consistently within a certain interval across different days (e.g., if meals are taken daily from 12 pm to 1 pm), it is considered a “hit”. Regularity in SRM is defined by the number of activities with three or more hits in 1 week; in our study, “activity” is considered to be the occurrence of the peaks and troughs of the probability of people making social connections. Again, we focus on within-individual variations. As shown in Fig. 3 in the main text, there are two peaks at 9 pm and 12 am for weekdays (8 pm and 12 am for weekends) and one trough at 8 am for weekdays (12 pm for weekends). We examined the peaks and troughs in each day during the 3-month period. If a peak or trough matches the general pattern (within an interval of 1 hour), then it is considered a “hit”. Table B below depicts the percentages of hits in the temporal occurrences of peaks and troughs for all days from January 1 to February 13.

**Fig. A. Distribution of the probability of making social connections.**

**Fig. B. Comparison between within-individual probability (WP), number of players online, number of players engaging in game activity (PVE), and the residual of WP on PVE.**

**Fig. C. Probability of making social connections by GDP level**

**Fig. D. Probability of making social connections by online purchase.**

**Fig. E. Probability of making social connections by game achievement levels.**

**Fig. F. Probability of making social connections by number of days being in the game after registration.**

**Fig. G. Probability of making social connections by number of friends.**

**Fig. H. Probability of making social connections by time zones.**

**Table A. List of Time Zone Divisions**

| **Zone** | **Provinces & Populations** | **Percentage of National Population** | **Percentage of Game Population** |
| --- | --- | --- | --- |
| GMT+9 | Northeastern China e.g., Jilin, Liaoning | 1.24% | 3.14% |
| GMT+8 | Eastern China (Coast) e.g., Beijing, Shanghai | 78.22% | 81.27% |
| GMT+7 | Middle China e.g., Guangxi, Hunan | 18.4% | 11.06% |
| GMT+6 | Western China e.g., Sichuan, Gansu | 1.98% | 1.49% |
| GMT+5 | Xinjiang and Tibet | 0.16% | 0.11% |

**Table B. Extent of “Hits” in the Temporal Occurrences of Peaks and Troughs (Jan. 1 to Feb 13).**

| **Date** | **Trough** | **Peak** | **Date** | **Trough** | **Peak** |
| --- | --- | --- | --- | --- | --- |
| Jan-01* | 0 | 21(hit) | Feb-14 | 6 | 0(hit) |
| Jan-02* | 7 | 20(hit) | Feb-15 | 8(hit) | 21(hit) |
| Jan-03 | 7(hit) | 22 | Feb-16 | 8(hit) | 0(hit) |
| Jan-04 | 8(hit) | 0(hit) | Feb-17 | 5 | 22 |
| Jan-09* | -- | -- | Feb-18 | 9(hit) | 0(hit) |
| Jan-10 | 5 | 9 | Feb-19* | 20 | 7 |
| Jan-11 | 8(hit) | 21(hit) | Feb-20* | 20 | 6 |
| Jan-12 | 9(hit) | 2 | Feb-21 | 20 | 6 |
| Jan-13 | 9(hit) | 2 | Feb-22 | 21 | 7 |
| Jan-14 | 7(hit) | 1(hit) | Feb-23 | 8(hit) | 7 |
| Jan-15* | 4 | 23(hit) | Feb-24 | 21 | 6 |
| Jan-16* | 9 | 20(hit) | Feb-25 | 21 | 7 |
| Jan-17 | 3 | 0(hit) | Feb-26* | 20 | 6 |
| Jan-18 | 8(hit) | 21(hit) | Feb-27* | 21 | 6 |
| Jan-19 | 8(hit) | 22 | Feb-28 | 14 | 6 |
| Jan-20 | 7(hit) | 0(hit) | Mar-01 | 23 | 0(hit) |
| Jan-21 | 8(hit) | 21(hit) | Mar-02 | 10 | 0(hit) |
| Jan-22* | 8 | 0(hit) | Mar-03 | 12 | 21(hit) |
| Jan-23* | 12(hit) | 20(hit) | Mar-04 | 12 | 2 |
| Jan-24 | 11 | 22 | Mar-11 | 12 | 3 |
| Jan-25 | 8(hit) | 20(hit) | Mar-12* | 12(hit) | 22 |
| Jan-26 | 6 | 2 | Mar-13* | 11(hit) | 20(hit) |
| Jan-27 | 7(hit) | 0(hit) | Mar-14 | 3 | 19(hit) |
| Jan-28 | 9(hit) | 0(hit) | Mar-15 | 13 | 22 |
| Jan-29* | 7 | 4 | Mar-16 | 9(hit) | 21(hit) |
| Jan-30* | 12(hit) | 6 | Mar-17 | 21 | 0(hit) |
| Jan-31 | 8(hit) | 20(hit) | Mar-18 | 0 | 21(hit) |
| Feb-01 | 0 | 22 | Mar-19* | 12(hit) | 22 |
| Feb-02 | 6 | 2 | Mar-20* | 11(hit) | 1(hit) |
| Feb-03 | 9(hit) | 4 | Mar-21 | 12 | 23(hit) |
| Feb-04 | 9(hit) | 23(hit) | Mar-22 | 11 | 0(hit) |
| Feb-05* | 10 | 0(hit) | Mar-23 | 12 | 1(hit) |
| Feb-06* | 9 | 3 | Mar-24 | 12 | 0(hit) |
| Feb-07 | 8(hit) | 20(hit) | Mar-25 | 0 | 23(hit) |
| Feb-08 | 8(hit) | 0(hit) | Mar-26* | 12(hit) | 3 |
| Feb-09 | 8(hit) | 2 | Mar-27* | -- | -- |
| Feb-10 | 14 | 4 | Mar-28 | 7(hit) | 11 |
| Feb-11 | 9(hit) | 23(hit) | Mar-29 | 8(hit) | 21(hit) |
| Feb-12* | 12(hit) | 4 | Mar-30 | 5 | 14 |
| Feb-13* | 8 | 3 | Mar-31 | 7(hit) | 20(hit) |

* indicates weekend.

*Note:*

*Weekdays: Trough = 8 a.m., Peak = 8 p.m. and 12 a.m.*

*Weekends: Trough = 12 p.m., Peak = 8 p.m. and 12 a.m.*

*Based on an interval of 1 hour, the following are considered “hits”:*

*Weekdays: Trough (7, 8, 9), Peak (19, 20, 21, 23, 0, 1).*

*Weekends: Trough (11, 12, 13), Peak (19, 20, 21, 23, 0, 1).*

*The following two days have no hits: Jan-09 has observations for 5 hours but all with zero probabilities; Mar-17 has observations for only 1 hour.*

1. The IP addresses are dynamic and only fixed to a specific area (i.e., non-individual specific). [↑](#footnote-ref-1)
