# Supplementary figures and images for: Circadian Rhythms in Socializing Propensity
Source: PLoS One. 2015 Sep 9;10(9):e0136325. doi: 10.1371/journal.pone.0136325 (PMC4564240; doi:10.1371/journal.pone.0136325)

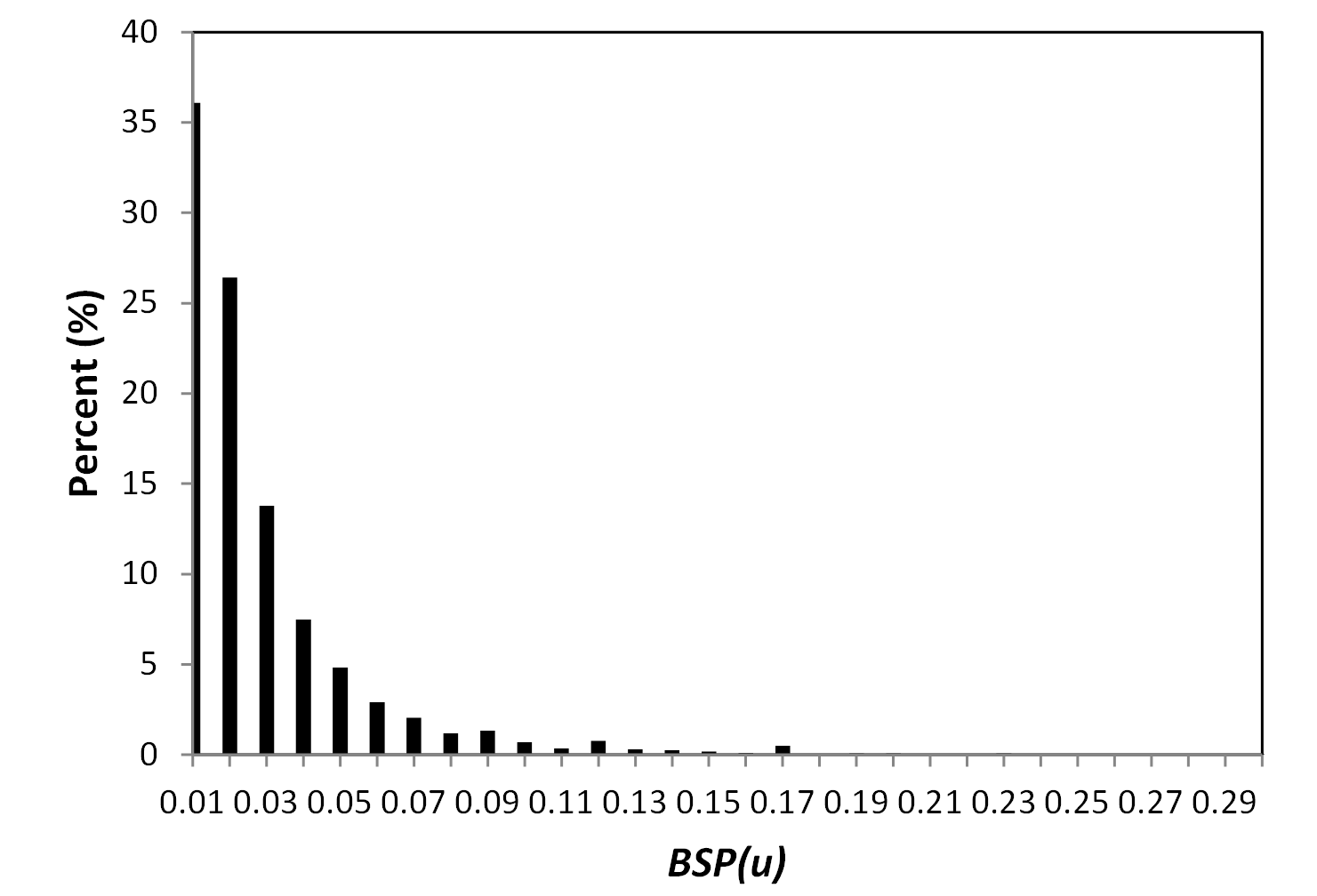

Supplement: S1 File — (ZIP) [file pone.0136325.s001.zip › S1 File/Fig.A.tif]

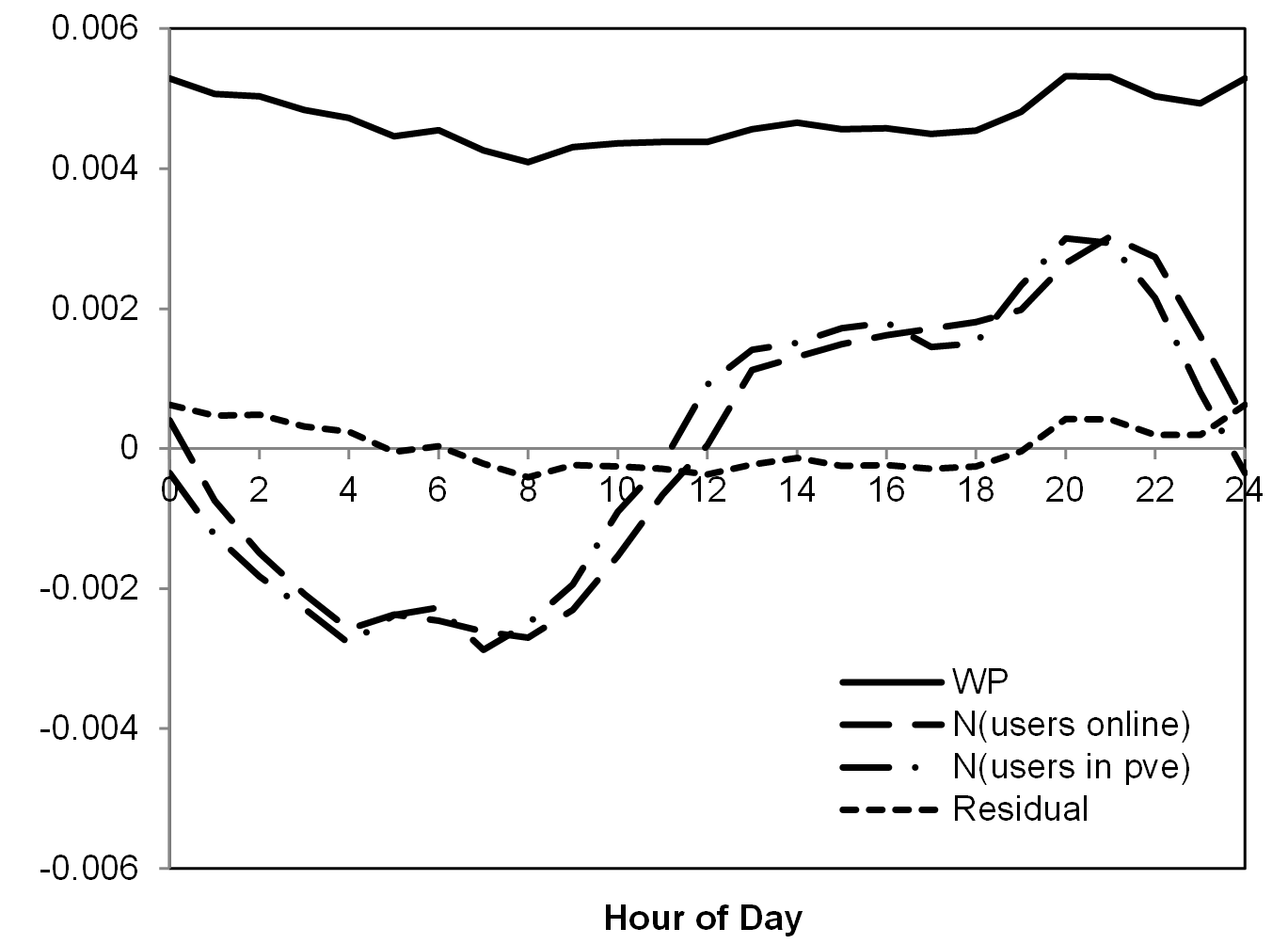

Supplement: S1 File — (ZIP) [file pone.0136325.s001.zip › S1 File/Fig.B.tif]

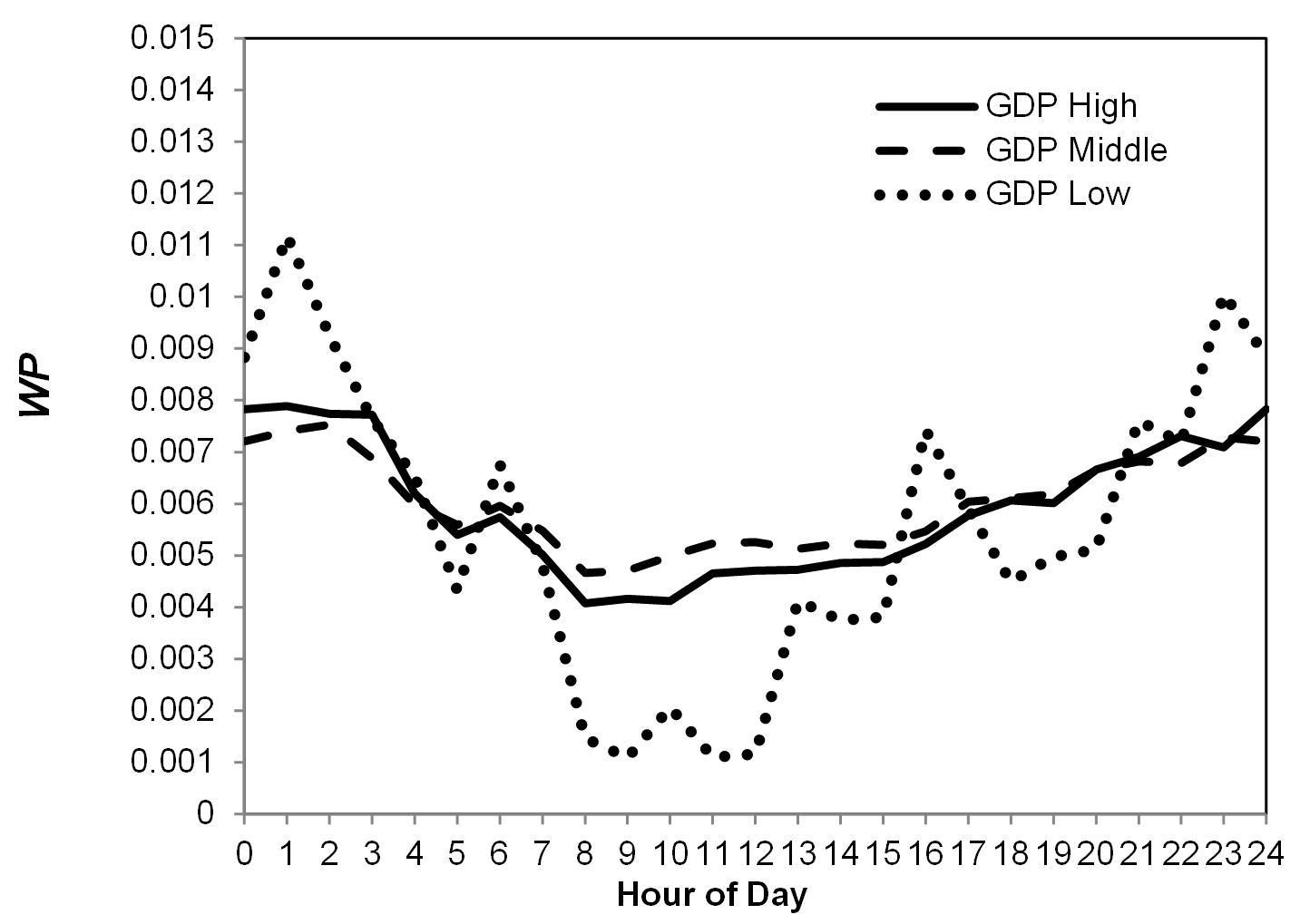

Supplement: S1 File — (ZIP) [file pone.0136325.s001.zip › S1 File/Fig.C.tif]

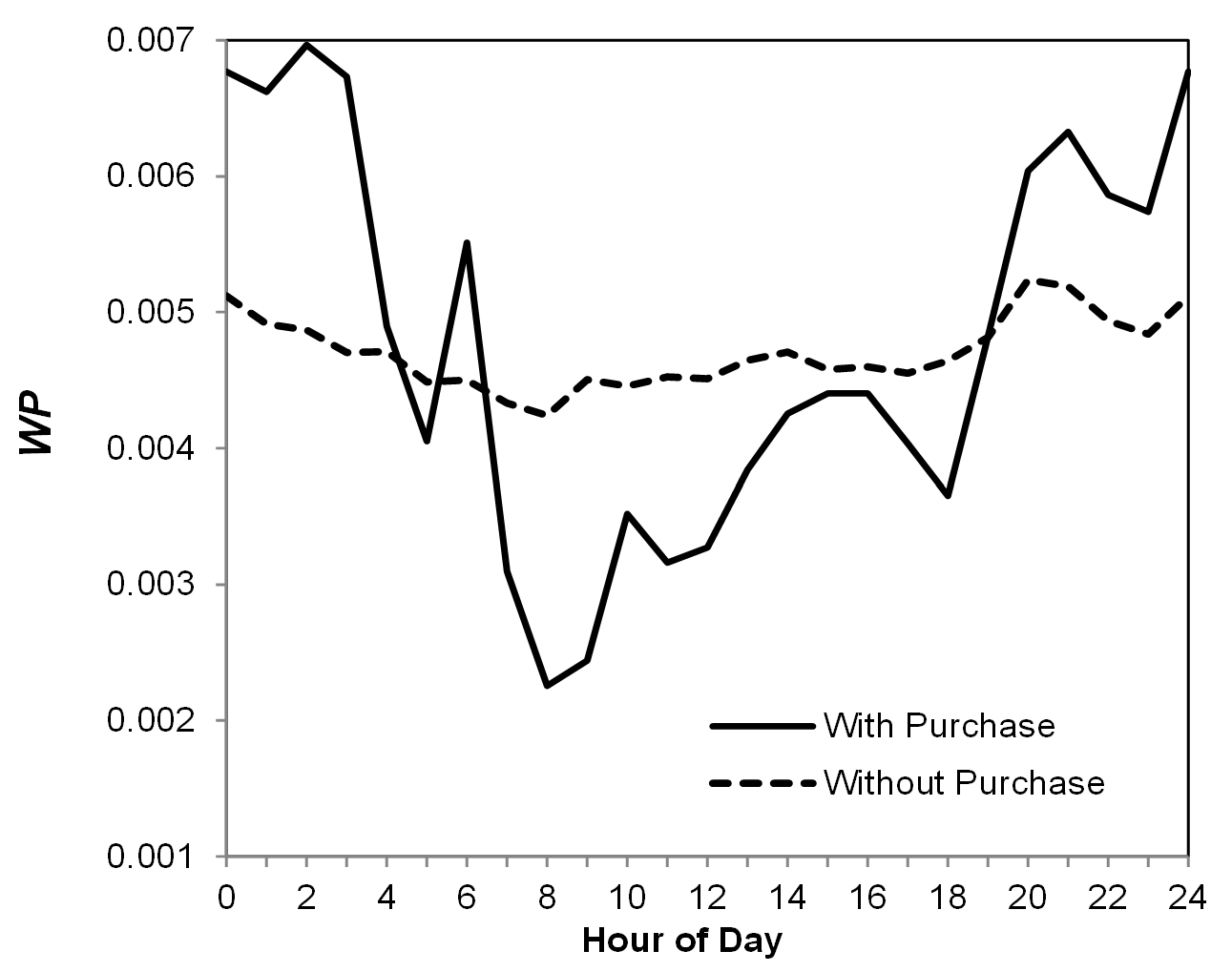

Supplement: S1 File — (ZIP) [file pone.0136325.s001.zip › S1 File/Fig.D.tif]

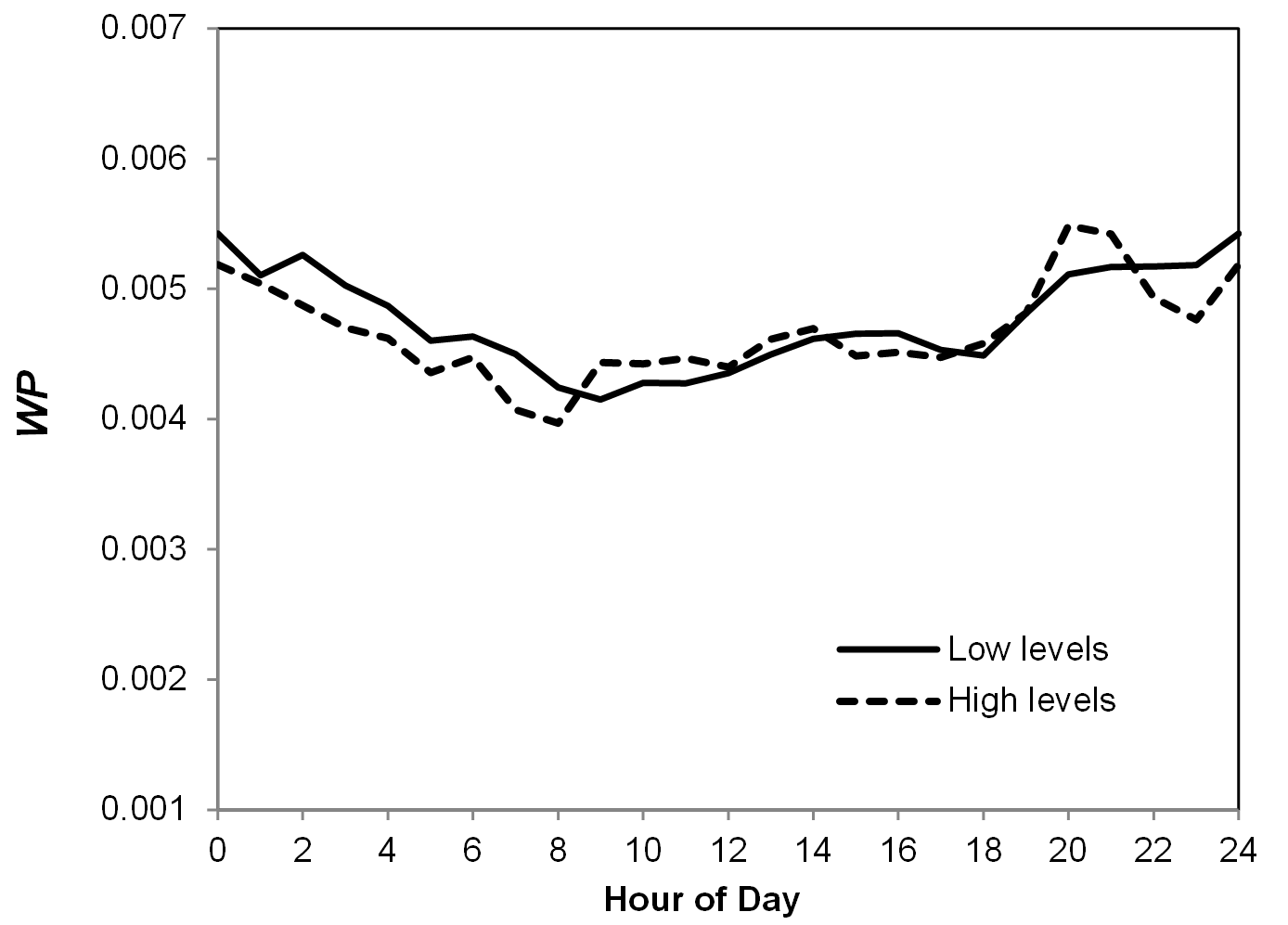

Supplement: S1 File — (ZIP) [file pone.0136325.s001.zip › S1 File/Fig.E.tif]

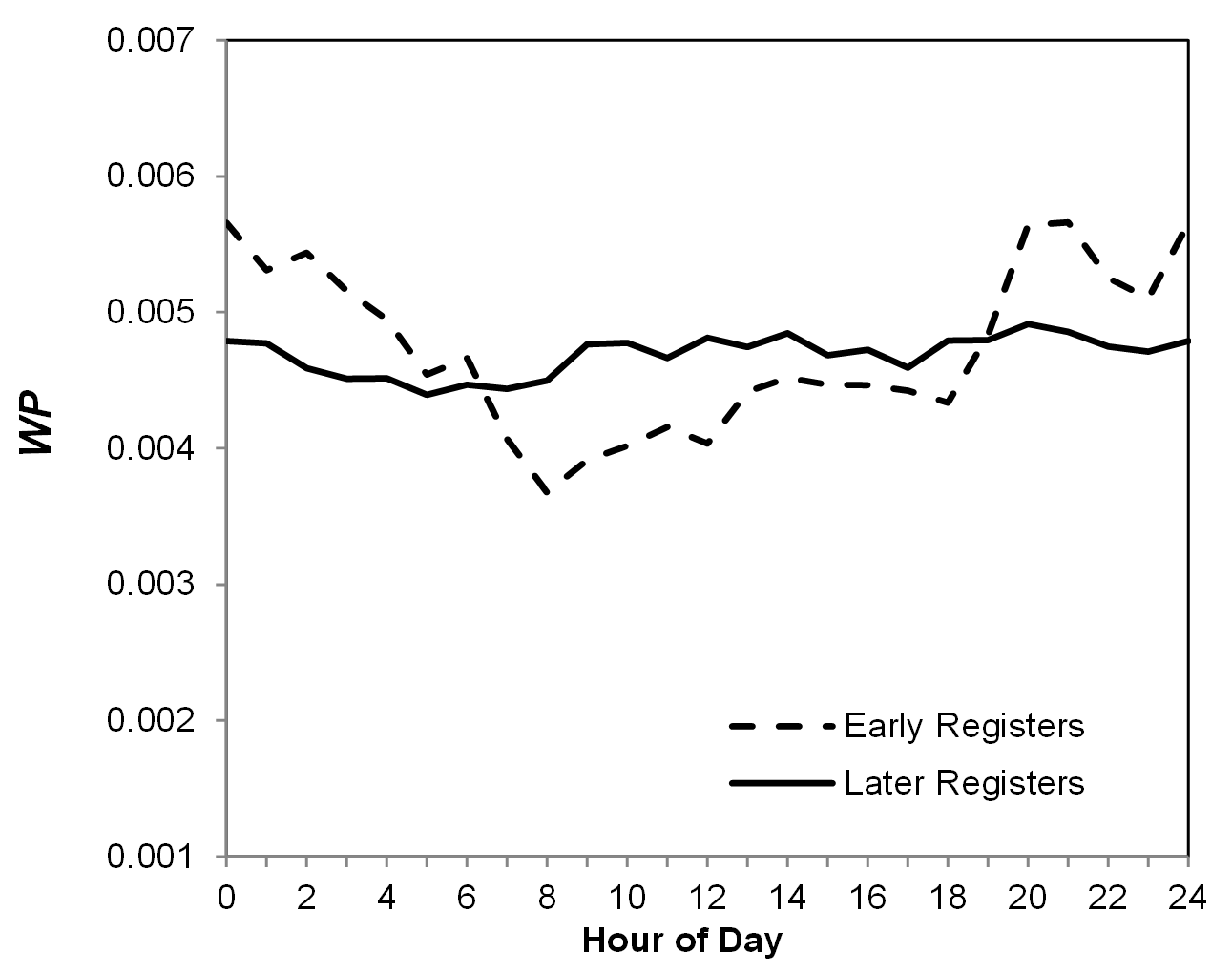

Supplement: S1 File — (ZIP) [file pone.0136325.s001.zip › S1 File/Fig.F.tif]

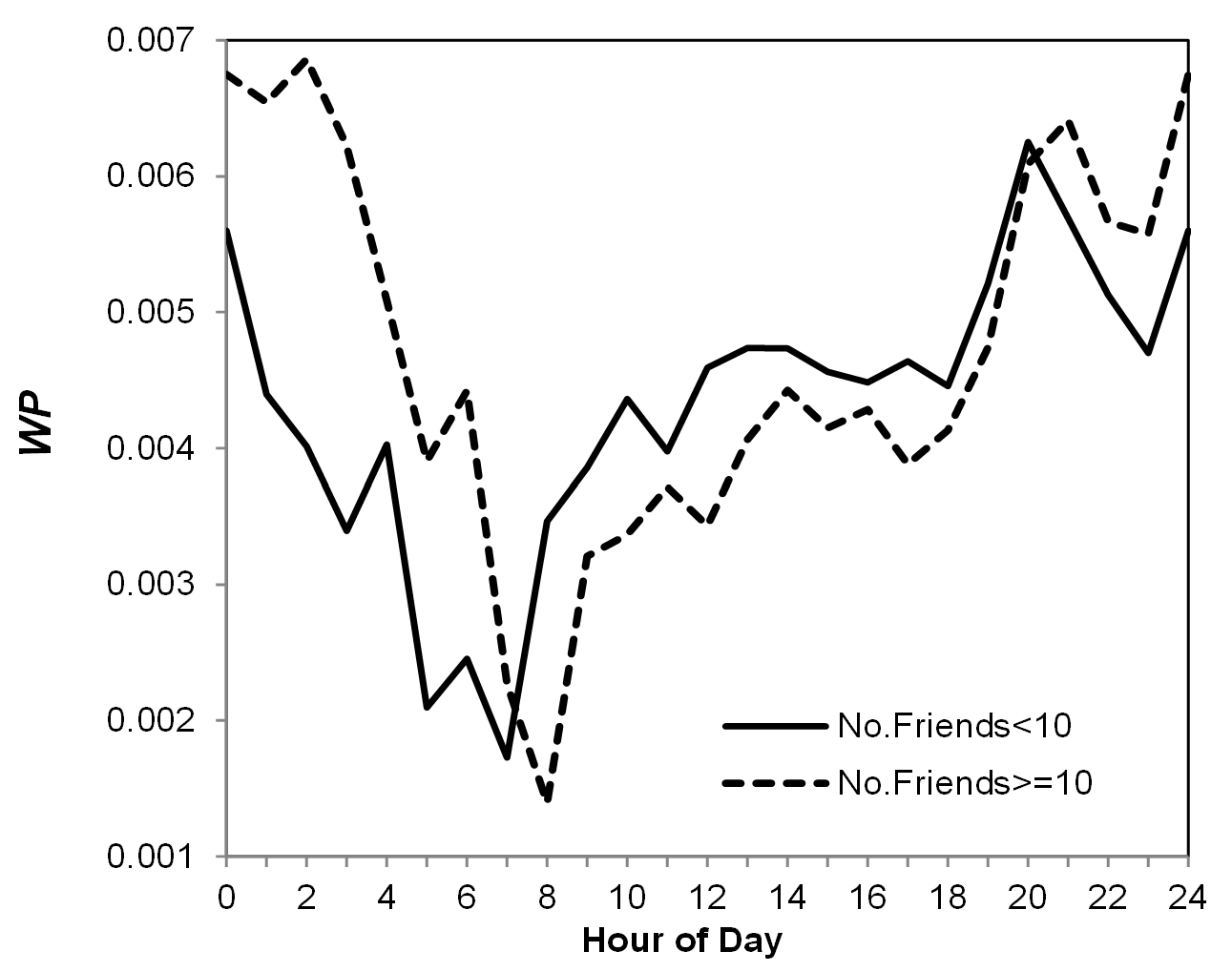

Supplement: S1 File — (ZIP) [file pone.0136325.s001.zip › S1 File/Fig.G.tif]

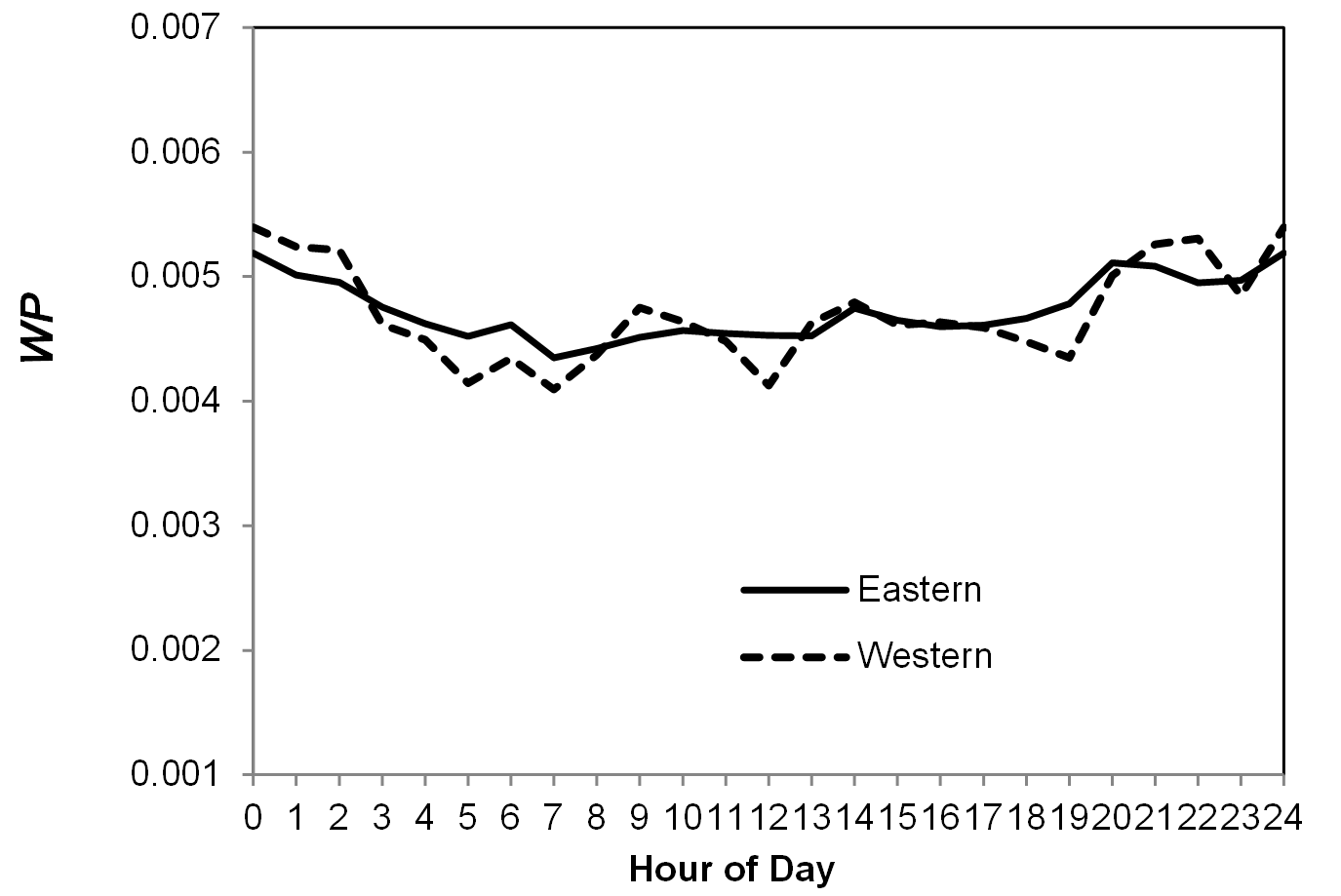

Supplement: S1 File — (ZIP) [file pone.0136325.s001.zip › S1 File/Fig.H.tif]
